# A Case of Medullary Exostosis

**Published:** 1849

**Authors:** E. C. Banks

**Affiliations:** Lawrenceville, Illinois


### ARTICLE VII.

*A Case of Medullary Exostosis.* By E. C. Banks, M. D. of Lawrenceville, Illinois.

Henry Thorn, a house carpenter, aged about 35 years applied to me on the 12th of October last, on account of a large tumor that occupied the entire calf of his right leg was very painful, and had continued to enlarge very fast, for some time. Although this tumo had been nine years and better in attaining its present size, he never suffered a particle of pain in it, since he discovered it, until about three months before I was summoned to attend him. I learned that about that time he fell and forced his leg in a flexed direction and hurt himself very much, and from this time he dates the commencement of his sufferings. Then, again, a few weeks afterwards, he struck his leg just between the tibia and fibula anteriorly, against a trussle bench and hurt it very much in that part, and from that a severe pain was felt and some enlargement took place at the precise spot where the injury was received. This enlargement has continued until it was, when I saw it, about the size of a hen's egg. His sufferings became so intense, that he could no longer work at his trade, and applied to me for relief. I prescribed a few moderate bleedings, blisters to the part, and followed these with hydriodate of potash internally, and tincture iodine, externally, with light bandaging to the part. I gave the hyd. potash, to try to correct the scrofulous habit of body that I believed to exist. I found that many of his relations had died of scrofulous disease in some form, and not a few with tubercular phthisis. Some relief was obtained for a few weeks, but then again his pains were as severe in the tumor and leg as ever; while his general health gave way. I advised amputation of the limb as the only means of cure, and even that under present circumstances, seemed doubtful. He and his friends readily agreed that the operation should be performed, and I set about preparing him for it. Amputation above the knee joint was the only chance, as the tumor grew from the superior posterior faces of the tibia and fibula.

Under the influence of chloroform, which completely prevented pain during the greater part of the time, the operation was performed on the 24th of November last.

I bled him next morning, as I had not allowed much hemorrhage to take place in the operation, and as high excitement seemed fast coming up. Since then, now near a month, he has been doing quite well. But a small part of the stump healed by the first intention.

*Character of the tumor.*—I have ventured to call what most writers would term osteo-sarcoma, by the name at the head of this article, as the tumor is more like brain than flesh.

I dissected off the muscles, and sawed through a bony shell, encasing a soft, brain-like substance, intermixed with spicula of bony matter, much softer and brittler than healthy bone. A soft, spongy, fragile, bony shell, surrounded the whole mass, except at its lower part, where it was soft, and when cut into, grumous blood flowed freely out, while the walls of the cavity containing it, resembled the membranes of the brain, though much thicker. The parts looked very like brain, intermixed with blood, and somewhat broken down. The bony shell surrounding the upper portion, and indeed nearly all the tumor was about as thick as ones hand, and resembled in shape a child's head with the occiput upward. It was also about the size of a young child's head, would weigh between four and six pounds, and was divided into several parts, by something resembling sutures, though only united by a strong membranous substance. There were four distinct pieces of bone united in that way forming the case to the tumor.

A part of the tumor came through between the tibia and fibula, growing, as it proved, from the anterior portion, crowding those bones apart considerably. The base of this was of the same bony substance, but the more superficial part contained bloody pus, and was soft to the touch. This he thinks was occasioned by the stroke against the corner of the trussle before spoken of. It was the most simple of any part. In the interstices of the different layers of bone, in this tumor, was a solid, matter, about the color and consistence of cheese.

